# In Vitro Evaluations and Comparison of the Efficacy of Two Commercial Products Containing Condensed Tannins and of Saifoin (*Onobrychis viciifolia* Scop.) Hay against Gastrointestinal Nematodes of Goats

**DOI:** 10.3390/ani13030547

**Published:** 2023-02-03

**Authors:** Alessia L. Gazzonis, Sara Panseri, Radmila Pavlovic, Sergio A. Zanzani, Luca Chiesa, Luca Rapetti, Marco Battelli, Luca Villa, Maria Teresa Manfredi

**Affiliations:** 1 Department of Veterinary Medicine and Animal Sciences, Università degli Studi di Milano, 26900 Lodi, Italy; 2 Proteomics and Metabolomics Facility (ProMeFa), IRCCS San Raffaele Scientific Institute, 20132 Milan, Italy; 3 Department of Agricultural and Environmental Sciences—Production, Landscape, Agroenergy, Università degli Studi di Milano, 20133 Milan, Italy

**Keywords:** condensed tannins, Eggs Hatch Test (EHT), gastrointestinal nematodes, Larval Migration Inhibition Test (LMIT)

## Abstract

**Simple Summary:**

Gastrointestinal nematodes (GINs) are a limiting health factor for dairy goat farming. To control them, the integration of the diet with bioactive fodder containing condensed tannins (CTs) was proposed by several authors. The present study aimed to both evaluate the in vitro anthelmintic efficacy of CTs contained in commercial products (Silvafeed BYPRO, SBP, and Silvafeed Q powder, SQ) and in sainfoin hay (SH) and carry out the untargeted metabolomics profiling of the selected formulations. This study provides useful information about the metabolomic profile of the ethanol and water extracts obtained using the analyzed formulations and a concentration that is effective on the egg hatching and migration ability of the infective larvae of goat GINs.

**Abstract:**

Gastrointestinal nematodes (GINs) is a limiting health factor for dairy goats, and the integration of the diet with fodder containing condensed tannins (CT) is becoming increasingly important to control GINs. To preliminary evaluate their potential role as part of GIN control in goat breeding, the in vitro anthelmintic efficacy of the CTs of Silvafeed BYPRO (SBP), Silvafeed Q powder (SQ), and sainfoin hay (SH) was evaluated, and the untargeted metabolomics profiling of the selected formulations was performed. CTs were extracted in water and in ethanol, their concentration was determined, and their chemical characterization was conducted using High-Performance Liquid Chromatography (HPLC) coupled to High-Resolution Mass Spectrometry (HRMS) platform. The in vitro anthelmintic activity of the extracts was evaluated using the Eggs Hatch Test (EHT) and the Larval Migration Inhibition Test (LMIT) using different extract concentrations (150, 300, 600, and 1200 μg/mL). The metabolomic profile of the ethanol extract showed a high number of flavonoids, while the water extract showed higher levels of hydrolysable tannins. The ethanol extracts were effective on both eggs hatching and larvae migration at low concentrations (150 μg/mL) for the three analyzed samples, while the water extracts showed more varied results: SH showed the greatest ovicidal efficacy (concentration 150 μg/mL, %IH = 40.9), while SBP and SQ were more effective against the larvae migration (concentration 600 μg/mL, %LMI = 69.7% and 88%), respectively. The integration of CT-rich fodder into the diet may be considered for the control of GIN infection in goats.

## 1. Introduction

Gastrointestinal nematodes (GINs) remain an important issue for livestock due to their repercussions on the health and welfare of animals. GINs are widely spread in small ruminants [1], also affecting productivity and therefore the farm economy even in the case of subclinical infections [2].

Control strategies currently rely on the administration of anthelmintic drugs; however, the systematic use of drugs for prophylactic purposes brings with it several problems, such as compliance with withdrawal times in meat and dairy animals, environmental pollution, and the possible onset of resistance to the most commonly used drugs, such as benzimidazoles and macrocyclic lactones. Indeed, anthelmintic resistance (AR) is a worldwide phenomenon that is more widespread in small ruminants than in cattle and horses due to the incorrect use of drugs [3]. In Italy, AR in small ruminants has already been reported in goats and, recently, in sheep [4,5]. This problem, combined with an increasing demand by consumers, who often prefer organic products, to reduce the use of drugs has prompted research to focus on the use of natural and traditionally grown fodder in the context of parasitic control strategies. 

Many plants traditionally cultivated or growing spontaneously in the areas intended for grazing animals have been studied for their use in the diet of small ruminants; in particular, plants rich in condensed tannins (CTs) have been the subject of numerous research studies for their anthelmintic activity [6]. In vitro and in vivo studies have showed the positive effect of the small ruminants of many plants rich in CTs, e.g., sainfoin (*Onobrychis viciifolia* Scop.), sericea lespedeza (*Lespedeza cuneata,* (Dum.Cours.) G. Don), lucerne (*Medicago sativa* L.), sulla (*Hedysarum coronarium* L.), and of commercially available purified CTs such as Quebracho [7,8,9,10,11,12], on gastrointestinal parasites. 

In northern Italy, most of the goat flocks graze from April to the end of October, and the goat’s diet is supplemented with fodder and concentrates depending on the intensity of grazing. Even if grazing has positive effects on the welfare of animals and the plant essences present in the grazing areas provide the cheeses with peculiar organoleptic characteristics, the pasture is a main risk factor for GIN infection. Indeed, high prevalence (76.6–100%) and abundance (1001–1498) values of GINs in grazing goats in northern Italy have been demonstrated [13,14].

Therefore, in the present work, the anthelmintic activity of a forage plant, sainfoin, which is commonly grown in Italy, and of two commercial products containing tannins was tested in vitro in order to obtain preliminary data on their potential use in the context of control strategies of GIN infections in goat breeding. In addition, chemical characterization based on High-Performance Liquid Chromatography (HPLC) coupled to a High-Resolution Mass Spectrometry (HRMS) platform was conducted to determine the untargeted metabolomics’ profiling to able to investigate the selected formulations. 

## 2. Materials and Methods

### 2.1. Plant and Products Collection

In vitro tests were performed on extracts from samples of about 100 g of two commercial products (Silvafeed BYPRO, SBP, and Silvafeed Q powder, SQ, by SILVATEAM, San Michele Mondovì, Cuneo, Italy) and of sainfoin hay (SH), characterized by different CT content and chemical composition. In particular, SBP is a premix formulated for ruminants containing condensed Quebracho tannin blended with hydrolyzable chestnut tannins and SQ is a feed additive with quebracho colorado condensed tannins extracted from *Schinopsis balansae* Engl. tree. Finally, SH used in the experiment was a representative sample of the hay produced in a field of 2.7 hectares located in Fiorenzuola (FI, Central Italy, 44°07′48.0″ N 11°20′01.0″ E).

### 2.2. Sample Preparation and Chemical Profiling

One gram of each sample was separately suspended in 100 mL of either water or ethanol (H_2_O- or EtOH- extracts, respectively). The samples were homogenized in the shaker for 24 h. An aliquot of 25 mL was dried using rotavapor and resuspended in 2 mL of initial HPLC mobile phase (95% water acidified with 0.1% HCOOH and 5% methanol). The resultant solution was diluted again in initial HPLC mobile phase (1:10) and 5 µL was injected. All samples were processed in two technical replicates. CT content was determined by using acetone butanol−HCl assay in SH and commercial products (SBP and SQ) [15].

#### HPLC—High-Resolution Mass-Spectrometric Profiling 

Chromatographical separation was accomplished using a Vanquish HPLC instrument (Thermo Fisher Scientific, San Jose, CA, USA) with a reversed phase column, Raptor ARC-18 (5 µm), 150 × 2.1 mm (Restek, Milan, Italy), with programed gradient flow of 0.1% HCOOH in water and methanol. The operative conditions were set up in order to achieve the best separation of the most important polyphenolic analytes. Exploris HRMS (Thermo Scientific, San Jose, CA, USA) operated in both positive mode and negative mode simultaneously, each one performed with predetermined acquisition parameters. The full scan (FS) with resolving power 120.000 (two scan range of *m/z* 70–800 and 800–2500) was used for the screening and statistical evaluation of the chromatographic profiles. Full scan data-dependent acquisition (FS-dd-MS2) with resolving power 60.000 and 17.500 for FS and dd-MS2, respectively, was employed for the fragmentation of pseudo-molecular ions detected in FS mode. 

An untargeted metabolomic workflow was applied as described with slight modifications [16]. Briefly, the Exploris Orbitrap raw data were submitted to Compound Discoverer (CD) 3.3 software (Thermo Fisher, MA, USA) that enabled the programmed compound’s identification and statistical evaluation. Differential analysis was performed as a part of the CD workflow and obtained statistical evaluation is expressed by hierarchical cluster processing, metabolic pathway schematization and box–whisker plots.

### 2.3. Recovery of GIN Eggs and Larvae for In Vitro Anthelmintic Assays

The anthelmintic activity of the three compounds was tested on eggs and third stage larvae (L3) of Strongylida. To obtain the eggs and L3, a fecal sampling from naturally infected goats bred in a dairy farm, located in the province of Varese, northern Italy, was carried out. Individual fecal samples were collected individually from the rectal ampoule of 15 adult goats. No anthelmintic treatment was administered to the animals in the three months prior to sample collection. The fecal samples were transported to the laboratory in refrigerated conditions within a few hours and processed immediately, as described below. Individual fecal samples were combined into a single pool for in vitro testing on Strongylida eggs and to obtain the L3 to be used for in vitro tests. All animal procedures used in this study were approved by the Milan University Institutional Animal Care and Use Committee.

#### 2.3.1. Egg Hatch Test (EHT)

The test was conducted following the procedures described in [17], with slight modifications. About 50 g of feces was diluted in water and then filtered through a mesh. The liquid was transferred into tubes and washed three times with water, centrifuging (4500 rpm, 5 min, 20 °C) and removing the supernatant. The obtained pellet was diluted in NaCl solution (specific gravity 1200) and centrifuged (4500 rpm, 5 min, 20 °C) twice. Each time, approximately 1 mL of the apical portion of the supernatant was withdrawn and placed in a new plastic tube; phosphate-buffered saline (PBS) was added and centrifuged to concentrate the eggs in the sediment. Finally, the eggs were examined using optical microscopy to assess that the embryonation process had not started. Then, the number of eggs present in the sample was determined, and the concentration was adjusted to 200 eggs/mL of the solution in PBS. The eggs were placed in a 24-well plate (100 eggs/well) and put in contact with the different dilutions in PBS of the products to be tested (obtaining the following dilutions of the products: 1200, 600, 300, and 150 mg/mL). Four replicates were prepared for each dilution. Negative (PBS) and positive (Thiabendazole 0.1 µg/mL) controls were added. After 48 h incubation in a thermostat at 25 °C and adequate humidity, the plate was read under an inverted microscope for the count of the first stage eggs and larvae. The percentage of the inhibition of hatchability (%IH) [18] was calculated for each well using the following formula: %IH = 100 × (1 − X1)/X2, where X1 = number of eggs hatched in contact with the extracts and X2 = number of eggs hatched in contact with the negative control.

#### 2.3.2. Larval Migration Inhibition Test (LMIT)

A fecal pool was processed to obtain the L3 to be used for the LMIT and for the identification of Strongylida at the genus level. L3 were obtained by preparing fecal cultures, incubating the fecal pool samples at 27 °C for 7 days, then recovered using the Baermann techniques and cleaned using a sucrose solution as previously described [19]. The obtained larvae were stored in water at 4 °C until analysis; an aliquot was used for morphological identification. The first 100 randomly selected L3 were identified to the generic level [20]. Larval culture showed that nematodes belonging to genera *Teladorsagia* (41.1%), *Oesophagostomum* (20.1%), *Haemonchus* (14.1%), *Trichostrongylus* (11.7%), *Cooperia* (8.2%), and *Chabertia* (4.8%) were the most common GINs which occurred in the fecal pool used for the in vitro tests. Before carrying out the LMIT, the harvest of the larvae was repeated using the Baermann technique; moreover, the viable larvae obtained were counted under optical microscopy, and the concentration was adjusted to 200 L3/mL of PBS. The test was conducted following the protocol described [19], with slight modifications. The L3 were placed in contact with different concentrations of CTs contained in the products to be tested (1200, 600, 300, and 150 mg of CT/mL of PBS). Four replicates were prepared for each dilution. Negative (PBS) and positive (Levamisole 1%) controls were added. In addition, a further control with the CT inhibitor polyvinylpolypyrrolidone (PVPP) (1200 mg of CT/mL of PBS for each product, diluted with 50 mg PVPP/mL of PBS) was tested in four replicates. After an incubation of 3 h in a thermostat at 20 °C, the samples were subjected to three washes with PBS, centrifuging (5000 rpm, 5 min) and removing the supernatant. Finally, each sample was allowed to migrate through a 20 µm diameter filter for 3 h at room temperature. Then, each sample was placed in the well of a 24-well plate, and the plate was read under an inverted microscope for the count of the migrated L3. The percentage of larval migration inhibition (%LMI) was calculated for each well using the following formula: %LMI = (T − M)/T * 100, where T = total number of tested larvae and M = number of L3 that have migrated.

### 2.4. Data Analysis

For %IH and %LMI, results were reported as mean ± standard error (s.e.). The differences between the means were analyzed using one-way analysis of variance (ANOVA) followed by Dunnett’s post hoc test (comparison of samples vs. negative control) or Tukey’s post hoc test (comparison between dilution with or without PVPP). The significance level was set at 0.05. Statistical analysis was performed using SPSS (version 19.0; SPSS, Chicago, IL, USA).

## 3. Results

The concentration of total CT was determined for each extract and the following results were obtained: 39.2 g/100 g in SBP, 68.3 g/100 g in SQ, and 2.1 g/100 g in SH. Based on these results, the working concentrations (1200, 600, 300, and 150 μg/mL) to be used in the in vitro parasitological study were prepared by diluting each extract in PBS.

**Figure 1:**
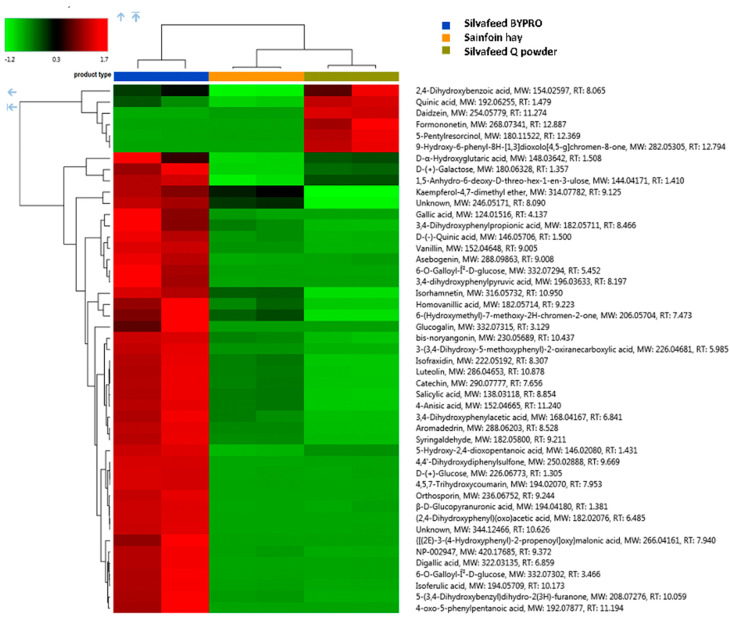
Hierarchical cluster analysis for the 45 most abundant species of flavonoids identified in ethanol extracts. Heatmap reflects the differences between the relative amount through normalized chromatographic peak areas in respect to sample type; z-color scale indicates normalized peak area value: red and green indicates more and less abundant, respectively.

**Figure 2:**
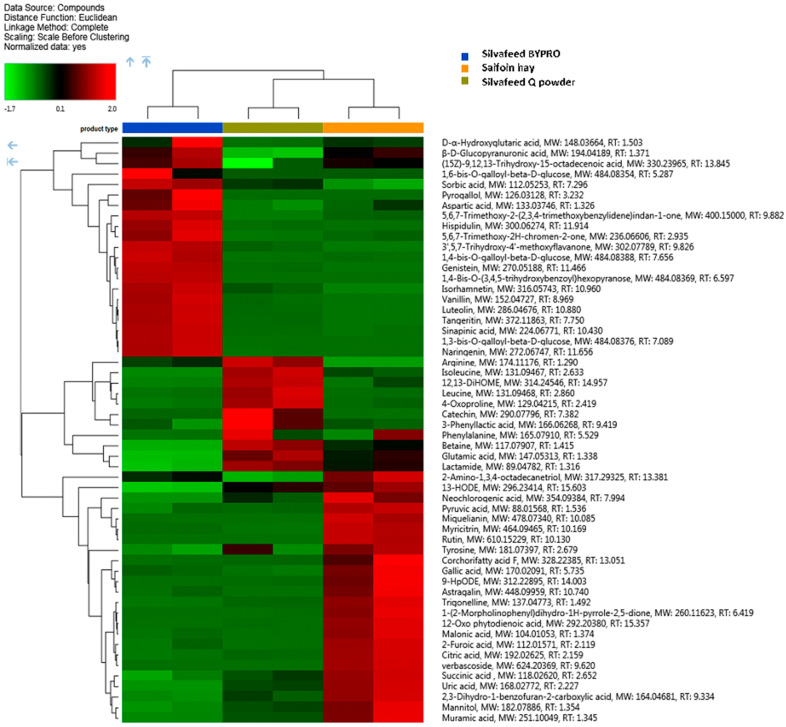
Hierarchical cluster analysis for the 55 most abundant species of hydrolysable tannins identified in water extracts. Heatmap reflecting the differences between relative amount through normalized chromatographic peak areas in respect to sample type; z-color scale indicates normalized peak area value: red and green indicates more and less abundant, respectively.

Regarding the metabolomic profiling (HRMS), overall, a total of 485 signals were individuated, from which the most abundant polyphenolic/antioxidant structures with a high level of confirmation certainty are presented in Figure 1 and Figure 2. The metabolomic profiles of the samples from two extraction protocols were different. The ethanol extract provided a high amount of some specific flavonoids, while the water extract was characterized by higher levels of hydrolysable tannins. For the ethanol extract, the flavonoid fraction that involves the formononetin derivates dominated in the SQ type (Figure S1). Furthermore, SBP samples were particularly rich in kaempferol-4.7-dimethyl ether, luteolin, and aromadendrin (Figure 1 and Figure 2), while SH had the highest content of essential amino acids such as arginine, leucine, isoleucine, and phenylalanine (Figure 2). Moreover, the most important finding of this investigation regards the hydrolysable tannins. Generally, hydrolysable tannins are characterized by the following models: (1) the structure contains galloyl units that are linked to diverse monosaccharide, catechin, or triterpenoid units; (2) the structure is formed by least two galloyl units C-C coupled to each other and does not contain a glycosidically linked catechin unit [21]. Besides a very low amount of free catechin that was detected either in the H_2_O or in EtOH extracts, the *m/z* signals that could be attributed to model 2 were scarce. On the other hand, a whole series of type 1 was characterized. Consequently, it was possible to reconstruct the hydrolysable tannins’ pathway with galloyl glucose derivatives as demonstrated in the Figure S2. Apart from different galloyl glucose forms, the presence of cornustannin 2 (also known as tellimagrandin I) should be emphasized. As shown in Figure S2, it is formed via the oxidation of pentagalloyl glucose and the enzyme pentagalloylglucose: O(2) oxidoreductase, a laccase-type phenol oxidase. Particular attention must be paid to the two isomeric ellagitannins that dominated in the SBP sample type, namely casuarinin and casuarictin (Figure S3).

**Figure 3:**
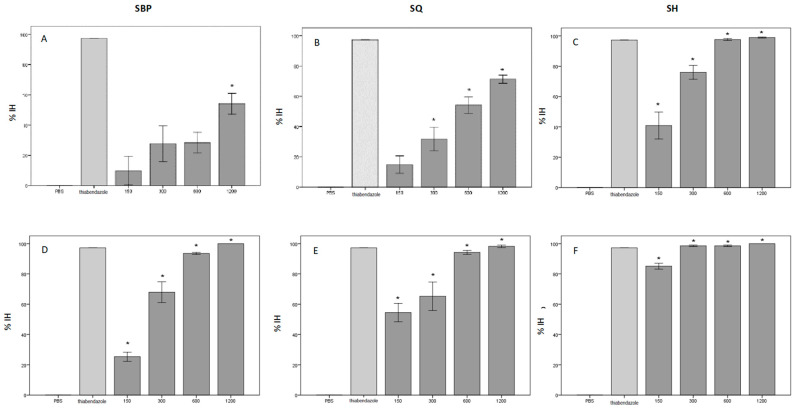
Percentage of inhibition of egg hatching with various concentrations of the tested extracts (%IH). Compared to phosphate buffer saline (PBS) and thiabendazole, %IH of PBS (negative control): 0%, %IH of thiabendazole (positive control): 97.3%. The significant inhibitory effects (*p*-value < 0.05, one-way analysis of variance, followed by Dunnett’s post hoc test) are indicated with *. Each bar represents mean ± standard error of the mean. (**A**–**C**) = H_2_O (water) extracts; (**D**–**F**) = EtOH (ethanol) extracts.

Considering the parasitological study, the results relating to the ovicidal capacity (EHT), as a consequence of the inhibition of the hatching of the eggs, of the CTs contained in the tested extracts are reported in Table 1 and graphically represented in Figure 3. Regarding the test conducted on the H_2_O-SBP extract, the effect on egg hatching inhibition was concentration-dependent, increasing the mean %IH from 9.8% (150 μg/mL) to 54.1% (1200 μg/mL); moreover, a statistically significant difference (*p*-value = 0.008) was recorded between the negative control and the H_2_O-SBP at the concentration of 1200 μg/mL, while the difference between the other concentrations tested and the negative control was not statistically significant. On the contrary, the EtOH-SBP extract seemed to be effective against eggs hatch starting from the first working dilution (150 μg/mL, *p*-value = 0.003), with a mean %IH of 25.3%. Similarly, considering the test conducted on Quebracho, the H_2_O-SQ extract showed efficacy on eggs hatching starting from the concentration of 300 μg/mL (mean %IH = 31.7%, *p*-value = 0.014), while the EtOH-SQ extract already starting from the first working concentration of 150 μg/mL, and the percentage of the inhibition of the egg hatchability (mean% IH = 54.4, *p*-value = 0.0001), was highest. Finally, the data relating to SH extracts showed a statistically significant efficacy starting from the concentration of 150 μg/mL both for the extract in H_2_O (mean% IH = 40.9, *p*-value = 0.001) and for that in EtOH (mean% IH = 85.1, *p*-value = 0.0001).

**Figure 4:**
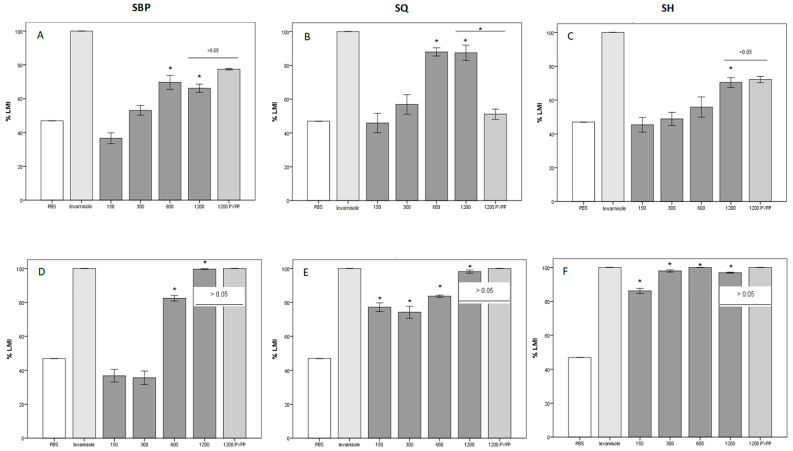
Percentage of inhibition of larval migration with various concentrations of the tested extracts (%LMI). Compared to phosphate buffer saline (PBS), levamisole, or polyvinylpolypyrrolidone (PVPP). %LMI of PBS (negative control): 47%, %LMI of levamisole (positive control): 100%. The significant inhibitory effects (*p*-value < 0.05, one-way analysis of variance, followed by Dunnett’s for PBS and by Tukey’s post hoc test for PVPP) are indicated with *. Each bar represents mean ± standard error of the mean. (**A**–**C**) = H_2_O (water) extracts; (**D**–**F**) = EtOH (ethanol) extracts.

The anthelmintic activity of the three products included in the study was subsequently tested on the L3 of Strongylida (LMIT). The data obtained during the test are reported in Table 2 and graphically represented in Figure 4. Considering the results obtained on the SBP, the anthelmintic effect of the aqueous and alcoholic extracts against L3 is effective starting from the concentration of 600 μg/mL (H_2_O-SBP: mean %LMI = 69.7%, *p*-value = 0.001; EtOH-SBP: mean %LMI = 82.5%, *p*-value = 0.0001). The aqueous extracts of SQ and SH were only effective starting from the concentration 600 μg/mL (H_2_O-SQ: %LMI = 88%, *p*-value = 0.0001) and 1200 (70.4, *p*-value = 0.013), respectively. On the contrary, alcoholic extracts proved effective in inhibiting the migration of L3 already at the first working concentration of 150 μg/mL (EtOH-SQ:%LMI = 77.2%, *p*-value = 0.0001; EtOH-SH:%LMI = 86.2%, *p*-value = 0.0001). The test also included a further control, adding to the maximum concentration (1200 μg/mL) of the three products tested using PVPP, a tannin inhibitor. Only for the aqueous extract of SQ did the mean %LMI decrease from 87.5% to 51.1% with the addition of PVPP (Tukey’s test: *p*-value = 0.001), while no difference was recorded for the other extracts.

## 4. Discussion

In recent years, in the context of strategies aimed at limiting the use of drugs used in the livestock sector, research has focused on the use of natural products. Among these, CTs showed, in addition to their various properties, anthelmintic activity, as has been highlighted in numerous studies conducted on the GINs of ruminants [7,8,9,10,11,12,22,23]. The need to use natural products derives on the one hand from the growing report of AR in small ruminants’ parasites worldwide [3] and, on the other hand, from the growing demand from consumers for food products derived from environmentally sustainable farming.

In the present study, a natural hay and two commercial products rich in CTs were evaluated in order to compare their chemical composition and anthelmintic efficacy. Two types of extraction were used, in ethanol and water, to investigate at a broad spectrum the analytes contained in the products under study.

Regarding the chemical characterization study, an HRMS-based metabolomics investigation was conducted for the first time in both polarization modalities (positive and negative) simultaneously within the same analytical run. In this manner, a reliable, sensitive, and double-confirmed identification of the key polyphenolic metabolites in the complex matrices of the three samples was achieved.

The metabolomic profile of the ethanol extracts showed a high number of flavonoids, while the water extracts showed higher levels of hydrolysable tannins. This first difference is reflected in the in vitro tests conducted to evaluate the efficacy of the extracts on the inhibition of Strongylida egg hatching and the migration activity of the larvae.

The ethanol extracts showed anthelmintic activity on both eggs hatching and larvae migration at low concentrations, with SQ and SH showing high levels of efficacy comparable to anthelmintic drugs (thiabendazole or levamisole). The results of the in vitro tests conducted on the extracts in the water instead show more varied results. The aqueous extract of SH showed the greatest ovicidal efficacy already at the first working dilution, while water extracts of SBP and SQ were more effective than SH against the larvae migration. Differences in the effectiveness of CT depending on the considered parasitic stages have already been reported [24]. Therefore, in order to determine the contribution of some bioactive compounds to the inhibition of larval migration, PVPP, forming complexes with tannins and polyphenols, and thus blocking their biological activity, was added to the tested extracts.

The obtained results suggest that the larvicidal activity of the extracts is totally attributable to tannins and polyphenols only in the case of the SQ extracted in the water. The exhibited anthelmintic extract seems attributed to the synergistic action of the components highlighted with chemical characterization. In the case of the other extracts, however, it appears that tannins and flavonoids were only partly involved in inhibiting the larval migration, and other biochemical compounds were also implicated.

The results obtained are in line with other in vitro studies. CTs extracted from sainfoin showed a 69% efficacy score in inhibiting eggs hatching at 200 μg/mL [25]. In another in vitro study, sainfoin extracts at 300–1200 μg/mL showed a significant inhibitory effect on the migration of *Haemonchus contortus* L3 [26]. Athanasiadou et al. [7], using the larval development/viability assay (LDVA), assessed the significant effect of Quebracho on the development and viability of nematode larvae varying among the investigated nematode species (*Teladorsagia circumcincta, H. contortus* and *Trichostrongylus vitrinus*).

The differences found in terms of anthelmintic efficacy against the hatching of the eggs or the migration of the larvae of the tested products also seem to depend on the parasitic taxon under study. Many studies have been conducted on *H. contortus*, which is a great model for in vitro studies due to its ability to survive in PBS [18], or on *Trichostrongylus colubriformis* and *Te. cirumcincta* [24]. However, the results may be poorly repeatable in caprine herds in which other GIN species predominate. For instance, in the farm used to obtain the eggs and larvae used for the in vitro tests carried out in this study, *H. contortus* represents only a small proportion of the identified GIN species. The choice was therefore to use eggs and larvae recovered from the fecal samples of naturally infected animals, and therefore those belonging to different parasitic taxa, in order to reproduce in vitro the real composition of the helminthofauna of goats bred in the study area [27].

The results obtained in this in vitro study can provide indications about the concentrations of CT-rich products and hay to be administered to animals in the context of GIN control strategies. However, it should be considered that in vivo the protein–tannin complexes may not be disrupted in the goat abomasum and that, consequently, the condensed tannins might be less effective on parasites. Translating to practice the results of the in vitro tests may be challenging, as the results may not exactly coincide with those obtained in vivo. Nevertheless, in vitro tests should always precede in vivo ones due to their cost-effectiveness, the time-saving benefits, and the reduction in animal use by protecting them from any toxic effects that could arise during the in vivo tests, in addition to useful indications for the use of in vivo bioactive products. Indeed, further perspectives include carrying out in vivo tests on dairy goats reared in a semi-intensive regime, particularly those exposed to GINs, with the aim of implementing the control of these parasites with the bioactive products showing the highest anthelmintic performances.

## 5. Conclusions

GINs are one of the major causes of economic losses in goat farming. With the need to intervene several times a year with the administration of anthelmintic treatments to control these parasites in grazing goats, and to reduce the phenomena of AR that could arise, different alternatives of natural origin are increasingly being used alongside the conventional drug. The in vitro tests carried out in this study have shown that the tested products appear to be good candidates to be used as a supplement to be included in the diet of goats to combat gastrointestinal parasites, thus contributing to a reduction in the use of anthelmintic drugs.

## Figures and Tables

**Table 1:** Descriptive data obtained with the egg hatch test (EHT) on the three products tested (extract concentrations: 150, 300, 600, and 1200 μg/mL, 4 replicates). The mean counts relating to the first stage larvae (L1) obtained for the replicates are reported. H_2_O: water extract; EtOH: ethanol extract; SBP: Silvafeed BYPRO; SQ: Silvafeed Quebracho powder; SH: Sainfoin hay; s.e.: standard error.

Extract Concentration (μg/mL)	SBP Extract		SQ Extract		SH Extract	
	H_2_O	EtOH	H_2_O	EtOH	H_2_O	EtOH
	mean ± s.e.	mean ± s.e.	mean ± s.e.	mean ± s.e.	mean ± s.e.	mean ± s.e.
150	9.8 ± 9.51	25.4 ± 3.03	14.9 ± 5.77	54.4 ± 6.07	40.9 ± 8.88	85.1 ± 2.00
300	27.7 ± 11.77	67.9 ± 6.87	31.8 ± 7.85	65.2 ± 9.41	76.0 ± 4.59	98.6 ± 0.55
600	28.4 ± 6.86	93.6 ± 0.65	54.1 ± 5.44	94.2 ± 1.28	97.6 ± 0.64	98.6 ± 0.55
1200	54.1 ± 6.91	100.0 ± 0.00	71.3 ± 2.73	98.3 ± 0.86	99.0 ± 0.35	100.0 ± 0.00

**Table 2:** Descriptive data obtained with the Larval Migration Inhibition Test (LMIT) on the three products tested (extract concentrations: 150, 300, 600, and 1200 μg/mL, 4 replicates) and on the concentration 1200 μg/mL with the addition of PVPP (tested in 3 replicates). The mean counts relating to the third stage larvae (L3) which actively migrated and those obtained for the replicates are reported. PVPP: polyvinylpolypyrrolidone; H_2_O: water extract; EtOH: ethanol extract; SBP: Silvafeed BYPRO; SQ: Silvafeed Quebracho powder; SH: Sainfoin hay; s.e.: standard error.

Extract Concentration (μg/mL)	SBP Extract		SQ Extract		SH Extract	
	H_2_O	EtOH	H_2_O	EtOH	H_2_O	EtOH
	mean ± s.e.	mean ± s.e.	mean ± s.e.	mean ± s.e.	mean ± s.e.	mean ± s.e.
150	36.6 ± 3.21	36.8 ± 3.72	45.9 ± 5.70	77.2 ± 2.61	45.4 ± 4.37	86.2 ± 1.50
300	53.1 ± 2.86	35.6 ± 3.98	56.9 ± 5.82	74.2 ± 3.56	48.9 ± 3.92	98.0 ± 0.64
600	69.7 ± 4.14	82.5 ± 1.68	88.0 ± 2.45	83.7 ± 0.63	55.9 ± 6.00	100.0 ± 0.00
1200	66.2 ± 2.45	99.7 ± 0.19	87.5 ± 4.53	98.2 ± 0.98	70.4 ± 2.95	97.0 ± 0.31
1200 with PVPP	77.4 ± 0.43	100.0 ± 0.00	51.1 ± 3.03	100.0 ± 0.00	72.2 ± 1.89	100.0 ± 0.00

## Data Availability

The datasets used and analyzed during the current study are available from the corresponding author on reasonable request.

## Supplementary Materials

The following are available online at https://www.mdpi.com/article/10.3390/ani13030547/s1. Figure S1. Pathway of formononetin derivates/metabolites with their relative amount presented through normalized chromatographic peak areas. The abbreviations SBP, SH, and SQ stand for sample type Silvafeed BYPRO, Sainfoin Hay, and Silvafeed Quebracho powder, respectively. Figure S2. Pathway of hydrolysable tannins’ interconnection with their relative amount presented through normalized chromatographic peak areas. The abbreviations SBP, SH, and SQ stand for sample type Silvafeed BYPRO, Sainfoin Hay, and Silvafeed Quebracho powder, respectively. Figure S3. box–whisker plots of two isomeric ellagitannins identified as predominant. Boxes represent 1st and 3rd quartiles, and median is indicated by the horizontal line inside the box; whiskers represent the upper and lower 25% of the distribution.
