# Supplementary material for: In Vitro Evaluations and Comparison of the Efficacy of Two Commercial Products Containing Condensed Tannins and of Saifoin (Onobrychis viciifolia Scop.) Hay against Gastrointestinal Nematodes of Goats
Source: Animals (Basel). 2023 Feb 3;13(3):547. doi: 10.3390/ani13030547 (PMC9913204; doi:10.3390/ani13030547)
Supplement: Supplementary file 1 [file animals-13-00547-s001.zip › animals-2174539-supplementary.pdf]

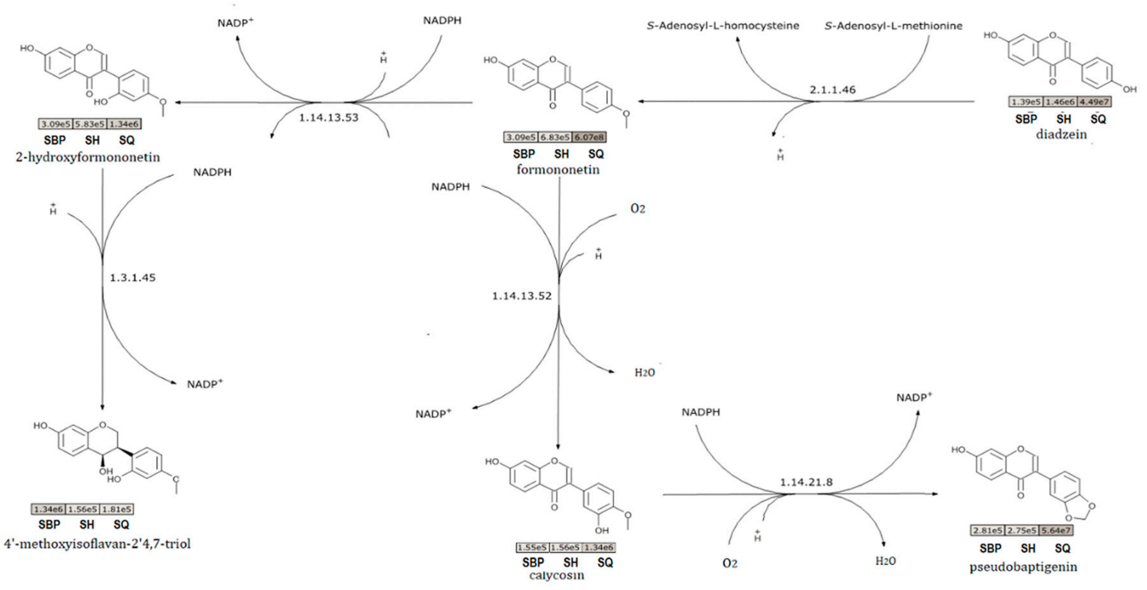

Figure S1. Pathway of formononetin derivates/metabolites with their relative amount presented through normalized chromatographic peak areas. The abbreviations SBP, SH, and SQ stand for sample type Silvafeed BYPRO, Sainfoin Hay, and Silvafeed Quebracho powder, respectively.

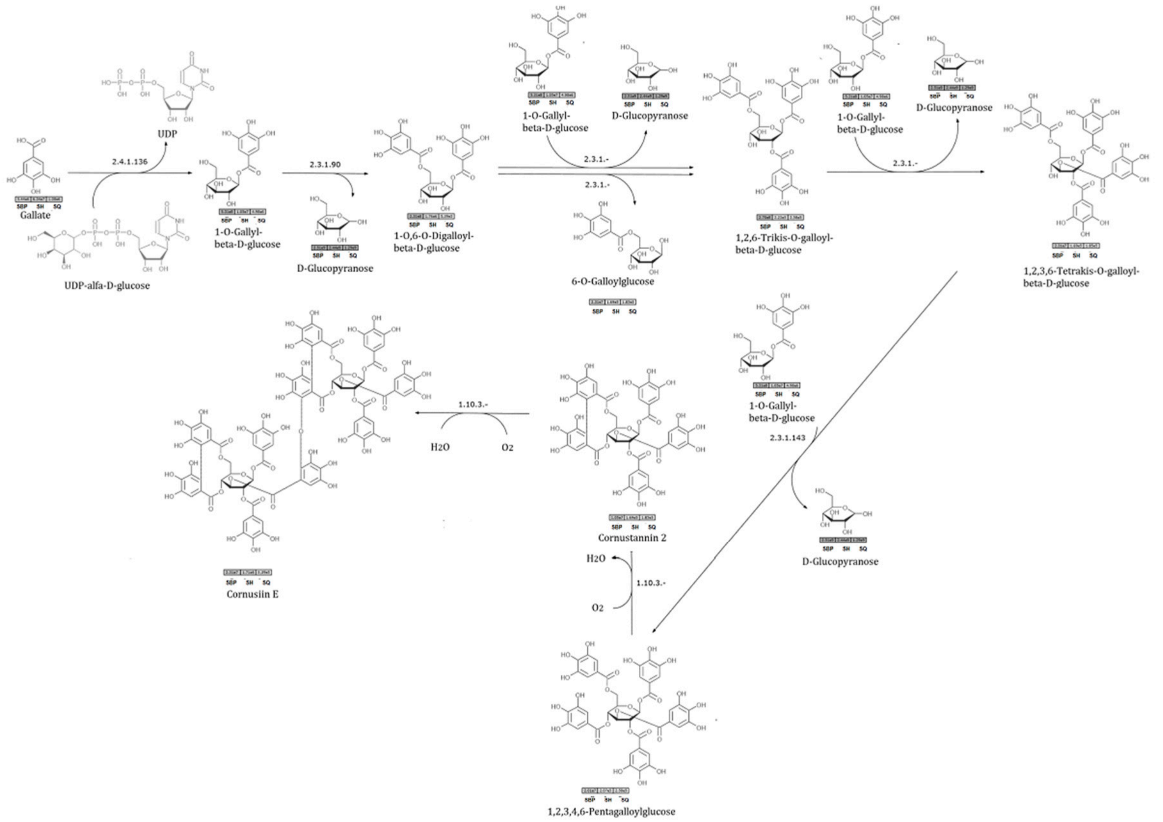

Figure S2. Pathway of hydrolysable tannins' interconnection with their relative amount presented through normalized chromatographic peak areas. The abbreviations SBP, SH, and SQ stand for sample type Silvafeed BYPRO, Sainfoin Hay, and Silvafeed Quebracho powder, respectively.

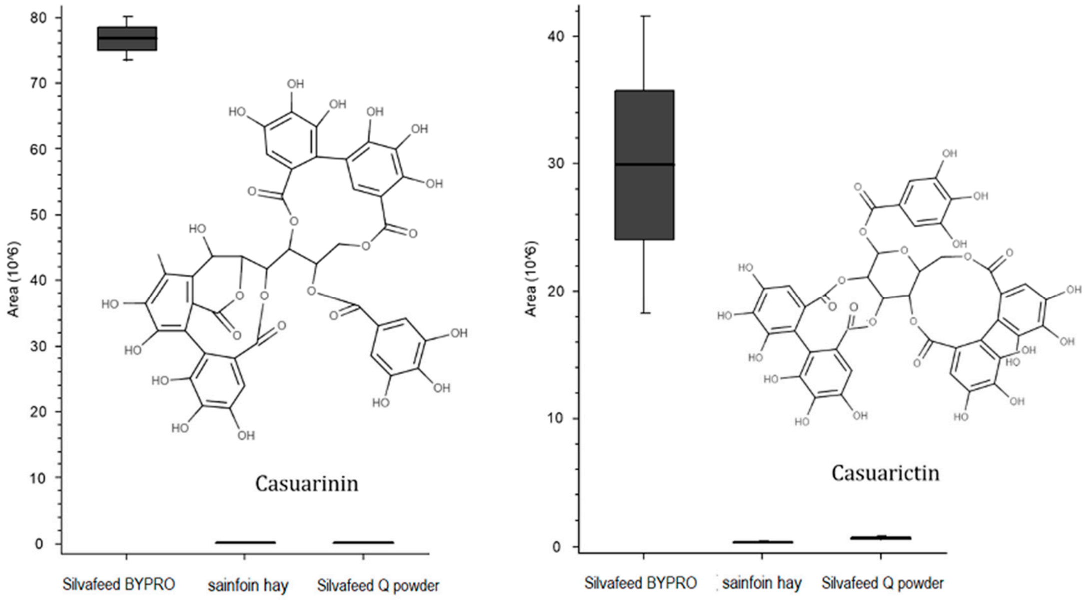

Figure S3. box–whisker plots of two isomeric ellagitannins identified as predominant. Boxes represent 1st and 3rd quartiles, and median is indicated by the horizontal line inside the box; whiskers represent the upper and lower 25% of the distribution.
